# ^109^Pd/^109m^Ag in-vivo generator in the form of nanoparticles for combined β^-^ - Auger electron therapy of hepatocellular carcinoma

**DOI:** 10.1186/s41181-024-00293-9

**Published:** 2024-08-13

**Authors:** Nasrin Abbasi Gharibkandi, Kamil Wawrowicz, Rafał Walczak, Agnieszka Majkowska-Pilip, Mateusz Wierzbicki, Aleksander Bilewicz

**Affiliations:** 1 Centre of Radiochemistry and Nuclear Chemistry, Institute of Nuclear Chemistry and Technology, Dorodna 16 St, Warsaw, 03-195 Poland; 2 Department of Medical Physics, M. Smoluchowski Institute of Physics, Faculty of Physics, Astronomy and Applied Computer Science, Jagiellonian University, Kraków, Poland; 3 Center for Theranostics, Jagiellonian University, Kraków, Poland; 4 Department of Nuclear Medicine, National Medical Institute of the Ministry of the Interior and Administration, Wołoska 137 St, Warsaw, 02-507 Poland; 5 Institute of Biology, Warsaw University of Life Sciences, Ciszewskiego 8 St, Warsaw, 02-786 Poland

**Keywords:** ^109^Pd/^109m^Ag in-vivo generator, Hepatocellular carcinoma, Auger electron therapy, Nanotechnology

## Abstract

**Background:**

Convenient therapeutic protocols for hepatocellular carcinoma (HCC) are often ineffective due to late diagnosis and high tumor heterogeneity, leading to poor long-term outcomes. However, recently performed studies suggest that using nanostructures in liver cancer treatment may improve therapeutic effects. Inorganic nanoparticles represent a unique material that tend to accumulate in the liver when introduced in-vivo. Typically, this is a major drawback that prevents the therapeutic use of nanoparticles in medicine. However, in HCC tumours, this may be advantageous because nanoparticles may accumulate in the target organ, where the leaky vasculature of HCC causes their accumulation in tumour cells *via* the EPR effect. On the other hand, recent studies have shown that combining low- and high-LET radiation emitted from the same radionuclide, such as ^161^Tb, can increase the effectiveness of radionuclide therapy. Therefore, to improve the efficacy of radionuclide therapy for hepatocellular carcinoma, we suggest utilizing radioactive palladium nanoparticles in the form of ^109^Pd/^109m^Ag in-vivo generator that simultaneously emits β^−^ particles and Auger electrons.

**Results:**

Palladium nanoparticles with a size of 5 nm were synthesized using ^109^Pd produced through neutron irradiation of natural palladium or enriched ^108^Pd. Unlike the ^109^Pd-cyclam complex, where the daughter radionuclide diffuses away from the molecules, ^109m^Ag remains within the nanoparticles after the decay of ^109^Pd. In vitro cell studies using radioactive ^109^Pd nanoparticles revealed that the nanoparticles accumulated inside cells, reaching around 50% total uptake. The ^109^Pd-PEG nanoparticles exhibited high cytotoxicity, even at low levels of radioactivity (6.25 MBq/mL), resulting in almost complete cell death at 25 MBq/mL. This cytotoxic effect was significantly greater than that of PdNPs labeled with β^−^ (^131^I) and Auger electron emitters (^125^I). The metabolic viability of HCC cells was found to be correlated with cell DNA DSBs. Also, successful radioconjugate anticancer activity was observed in three-dimensional tumor spheroids, resulting in a significant treatment response.

**Conclusion:**

The results indicate that nanoparticles labeled with ^109^Pd can be effectively used for combined β^−^ - Auger electron-targeted radionuclide therapy of HCC. Due to the decay of both components (β^−^ and Auger electrons), the ^109^Pd/^109m^Ag in-vivo generator presents a unique potential in this field.

## Background

Hepatocellular carcinoma (HCC) is the fifth most common cancer and one of the three deadliest cancers worldwide (Torre et al. 2015). Despite considerable progress in cancer therapy, such as targeted and immunotherapies, liver transplantation is still the most efficient option to prolong the life quality in patients with HCC. Surgical resection, radiofrequency ablation, transarterial chemoembolization, radioembolization, and combination approaches are approved treatments for advanced HCC. However, these modalities do not significantly extend life expectancy or prevent disease recurrence (Medavaram and Zhang 2018). Another option - chemotherapy involves drug infusion into the hepatic artery, which is designed to limit side effects to the rest of the body (Chakraborty and Sarkar 2022). Once delivered in this way, a significant dosage of anticancer drugs will be broken down by the healthy liver cells. Although the overall effects of the chemotherapeutic drugs have decreased, some treatment-associated adverse effects still persist, such as hair loss, nausea, and fatigue (Medavaram and Zhang 2018). Additional therapy commonly used for advanced HCC includes broad-spectrum tyrosine kinase inhibitors, such as sorafenib and lenvatinib, as well as a combination of immunotherapy and anti-angiogenesis therapy (Fu and Wang 2018; Greten and Sangro 2018). These strategies offer a nominal extension of the survival curve, but these improvements can be measured in months and result in widely observed toxic side effects, ultimately leading to patients developing resistance to therapy. For this reason, there is a a significant need to design novel therapy options for liver cancer patients who cannot undergo surgical treatment.

Many researchers have shifted their focus to the field of nanotechnology with the aim of addressing this issue. In a review article, Mintz and Leblanc (Mintz and Leblanc 2021) presented over a hundred studies on the possibility of using nanostructures for liver cancer therapy. According to Web of Science, only in 2023, 567 papers containing the keywords “nanoparticles” and “liver cancer” were published. The studies were conducted with both inorganic nanoparticles and carbon-based nanostructures, where the role of nanostructures was either as a drug carrier or the nanoparticles themselves were therapeutic.

The application of nanostructures in liver cancer treatment offers significant advantages, such as reducing therapy-related toxicity and increasing the possibility of precise drug delivery. The common feature of nanoparticles is accumulation in the liver after systemic injection, but this phenomenon depends highly on their properties (Blanco et al. 2015). This remains valid for the majority of nanoparticles, mainly inorganic; however depending on size and charge, some may accumulate in the kidneys and spleen as well (Demoy et al. 1999). As commonly known, blood from the circulation reaches the liver before reaching the kidneys, but not all of the blood supply passes through the liver. Accumulation in the kidneys can be prevented by using negatively charged very small nanoparticles with a hydrodynamic diameter of 6–8 nm, which are not filtered by the kidneys and pass to the liver, where their accumulation is observed (Mintz and Leblanc 2021). Specificity to cancer cells in the liver can be ensured through passive targeting by utilizing the enhanced permeability and retention (EPR) effect. Due to the EPR effect, small-sized nanoparticle drugs can accumulate more effectively in the tumor than in healthy tissues (Yhee et al. 2013). This phenomenon is possible due to the leaky tumor vasculature through which nanostructures can leave the bloodstream, pass through the gaps in the vessels’ endothelial lining, and enter the tumors.

A variety of nanoparticle types, including various metallic ones, have been utilized for the treatment of HCC. Among the ranges studied, platinum nanoparticles proved to be the most advantageous. The use of platinum nanoparticles in cancer therapy seems logical, considering the extensive application of cisplatin in cancer therapy. For instance, Medhat et al. (Medhat et al. 2017) reported that platinum nanoparticles displayed an IC50 value of 10.3 µM, whereas cisplatin showed a value of 26.5 µM, indicating increased chemotherapeutic efficacy of platinum nanoparticles. Moreover, the measured parameters for liver function were notably closer to the control group for platinum nanoparticles compared to cisplatin, indicating reduced side effects.

The main inspiration for our studies described in the present publication was the paper by Wennemers et al. (Shoshan et al. 2019) on the selective toxicity of small platinum nanoparticles against hepatocellular carcinoma cells. The authors explained the strong and selective cytotoxic effect by intracellular oxidation of Pt^0^ to Pt^2+^ followed by the release of Pt^2+^ ions from nanoparticles that block cell division by binding to DNA, inducing DNA damage. Therefore, PtNPs are expected to cause substantially higher toxicity in cells with a high oxidation state, thus being highly selective and safe for healthy tissues. Taking into account the redox potential of the reaction Pt^2+^ + 2e → Pt^0^ = 1.18 V, the oxidation of metallic platinum in H_2_O_2_ solutions cannot be expected. However, as was found in the case of noble metal nanoparticles, small nanoparticles, and metal clusters show significantly greater reactivity in redox (e.g. with H_2_O_2_) systems than in bigger block structures (Ye et al. 2016).

Since platinum has two radioisotopes, ^193m^Pt and ^195m^Pt, which are Auger electron emitters, nanoparticles synthesized with these radionuclides should have a multiplied cytotoxic effect. It was found in the metastatic tumor cell studies that both the non-radioactive Pt and ^195m^Pt complexes had a considerable therapeutic anticancer effect (expressed as DNA damage in tumor cells). Notably, it was 11-fold higher when Auger-emitting ^195m^Pt was used instead of non-radioactive Pt (Nadar et al. 2021). As low-energy Auger electrons exhibit high toxicity only upon intercalation into DNA strands (Aghevlian et al. 2017; Ku et al. 2019), in the case of ^193m,195m^Pt nanoparticles, this effect should specifically affect cells with a high concentration of H_2_O_2_, such as HepG2, whereas postulated (Shoshan et al. 2019) Pt nanoparticles can undergo at least partial dissolution. Consequently, a high selectivity of cytotoxic effects can be achieved. Unfortunately, our recent studies have clearly demonstrated that it is basically impossible to generate sufficient activity of ^193m,195m^Pt for application in Auger electron therapy (Wawrowicz and Bilewicz 2023). Due to the expected minimal bone marrow and normal tissue toxicity, very high activities of the radioisotope per patient are administered in targeted Auger radionuclide therapy. For instance, in clinical studies concerning neuroendocrine tumour therapy, patients have received accumulative ^111^In radioactivity of up to 100 GBq to boost the therapeutic response without any notable side effects (Kwekkeboom et al. 2003). Hence, it can be concluded that it is currently impractical to obtain such activities of ^193m,195m^Pt to perform Auger electron therapy, as we discussed previously (Wawrowicz and Bilewicz 2023) .

Due to the similar *d*^8^ electron configurations and the high chemical similarity of Pt^2+^ and Pd^2+^ cations, palladium nanoparticles are interesting candidates for Auger electron therapy. This includes possible solubility in higher H_2_O_2_ concentrations. Furthermore, many publications have confirmed the anti-cancer properties of Pd^2+^ cations and their complexes. As a result, the two palladium radionuclides,^103^Pd and ^109^Pd, can serve as great alternatives for ^193 m,195m^Pt. Palladium-103, due to the emission of X-ray radiation in the 20–23 keV range, is widely used in seed form for prostate brachytherapy Therefore the methods of its production on both research and industrial scale have already been developed. This radionuclide can be produced by thermal neutron irradiation of the isotopically-enriched ^102^Pd target in the nuclear reactor or by proton irradiation of ^103^Rh monoisotopic target. Although ^103^Pd emits a limited number of Auger electrons, its decay product − ^103m^Rh is one of the most promising candidates for Auger electron radiotherapy, according to the reaction (Filosofov et al. 2021; Peter Bernhardt 2001) .

$$^{103}Pd^{\:\:\underrightarrow{EC\left(branching\:ratio\:99.97\%\right)}}\:\:\:^{103m}Rh^{\underrightarrow{IT,\:\:\:Auger\:electrons}}\,{^{103}}Rh$$

Unfortunately, the short half-life of ^103m^Rh (t_½_=56.11 min) causes challenges and makes it almost impracticable to prepare the ^103m^Rh radiopharmaceuticals. Nevertheless, it can serve as an in-vivo generator of ^103^Pd/^103m^Rh for targeted therapy.

Second palladium radionuclide − ^109^Pd, has excellent potential for use in radionuclide therapy. Palladium-109 undergoes β^−^ decay (β_max_ = 1.12 MeV, 100% yield) to ^109m^Ag (t_½_=39.6 s). The formed metastable ^109m^Ag decays to stable isotope ^109^Ag which is accompanied by photon emission of 88-keV(3.6%), followed by cascade emission of both conversion and Auger electrons. Such properties enable its simultaneous application in both low- and high-LET internal radiation therapy. This approach relies on the simultaneous destruction of large tumors using β^−^ radiation while additionally improving the treatment for tumor subpopulations, including resistant cancer stem cells or small metastases by either Auger electrons or α emitters (Stokke et al. 2022). Recently published groundbreaking studies by Mueller et al. (Müller et al. 2019) have demonstrated that the utilization of ^161^Tb can lead to a significantly greater therapeutic effect compared to similar studies involving ^177^Lu. This distinctive feature of ^161^Tb is based on the simultaneous emission of β^−^ (β_max_ ~ 550 keV) and Auger electrons (12.1 e^−^ per decay), which prevails over the nearly pure β^−^ emitter ^177^Lu (β_max_ = 497 keV) followed by only a minimal number of Auger/conversion electrons (~ 1.11 electrons per decay) (Müller et al. 2019) .

In this study, we propose the utilization of ^109^Pd in the form of a ^109^Pd/^109m^Ag in-vivo generator as an alternative to ^161^Tb produced via the indirect route by neutron irradiation of ^160^Gd. The proposed approach involves the application of ^109^Pd/^109m^Ag in-vivo generators, which offer advantages over ^161^Tb due to a greater number of Auger/conversion electrons emission (18 vs. 12.1). Furthermore, in contrast to ^161^Tb, ^109^Pd can be simply produced by thermal neutron irradiation. Activation of the enriched metallic Pd target (98% in ^108^Pd) with a thermal neutron flux of 3 × 10^13^ n cm^− 2^ s^− 1^ for three days results in a specific activity of 1.85 GBq/mg and almost 100% radionuclide purity (Das et al. 2008). A high-flux reactor (> 10^15^ n cm^− 2^ s^− 1^) can boost this yield, increasing the specific activity up to 40 GBq/mg. Contrary to ^161^Tb, palladium, due to its chemistry, cannot be used in the form of complexes with the most commonly used chelators, thus its application in NP-based radioconjugates makes it especially convenient to liver cancer therapy.

In previous work, we presented the results of our studies on the use of a radiobioconjugate of gold nanoparticles covered with a ^109^Pd layer and attached to the monoclonal antibody trastuzumab (Gharibkandi et al. 2023). The positive results obtained encouraged us to continue our studies oriented at hepatocellular carcinoma (HCC) therapy with small pegylated radioactive ^109^Pd nanoparticles. Due to the anticipated dissolution of nanoparticles in HepG2 cells containing elevated levels of H_2_O_2_, we expected a significant cytotoxic effect. Additionally, our study aimed to deeper investigate whether the radionuclide ^109m^Ag formed during the decay of ^109^Pd remains within the palladium nanoparticles or diffuses away from them.

## Materials and methods

### Reagents

The used chemical reagents include: Palladium(II) chloride, (HS–PEG–COOH (poly(ethylene glycol), 5 kDa) from Sigma-Aldrich (St. Louis, MO, USA). Enriched palladium ^108^Pd (> 99%) was acquired from Isoflex (San Francisco, CA USA). Hydrochloric acid, sodium hydroxide, and hydrogen peroxide 30% were purchased from POCH (Gliwice, Poland).

The following materials were utilized in cell studies: MEM-EAGLE medium, trypsin EDTA solution C, fetal bovine serum (FBS) from Biological Industries (Beth Haemek, Israel); phosphate-buffered saline (PBS), w/o calcium and magnesium, dimethylsulfoxide (DMSO), and the CellTiter 96^®^ Aqueous One Solution Reagent (MTS compound) from Promega (Mannheim, Germany). HepG2 cells were obtained from the American Type Tissue Culture Collection (ATCC, Rockville, MD, USA) and cultured following the ATCC protocol. For experimental applications, over 80% confluent cells were used. Cells were maintained in MEM-EAGLE medium enriched with 10% heat-inactivated fetal bovine serum, 1% L-glutamine (200 mM), and antibiotics (penicillin 100 IU/mL and streptomycin 100 µg/mL).

Anti-phospho-Histone H2A.X (Ser139) antibody, clone JBW301; anti-mouse IgG (H + L), CF™ 633 antibody produced in goat were used to prepare samples for DNA double strand breaks study. Other reagents for DNA double strand breaks study included 4% paraformaldehyde (PFA) in PBS as fixative, bovine serum albumin (BSA), Triton X-100, and TBS (tris buffered saline) purchased from Merck & Co., Inc. (Kenilworth, NJ, USA). DAKO Fluorescent Mounting Medium was obtained from Agilent Technologies (Santa Clara, CA, USA). For DNA staining, Hoechst 33,258, purchased from Thermo Fischer Scientific (Waltham, MA, USA), was used. All solutions were prepared using double-distilled water (18.2 MΩ·cm, Hydrolab, Straszyn, Poland).

### Instruments

The size and morphology of nanoparticles were examined using a Zeiss Libra 120 Plus TEM operating at 120 kV (Zeiss, Stuttgart, Germany). The DLS method was used to analyze the hydrodynamic size of the synthesized nanoparticles and their conjugates with PEG. The hydrodynamic diameter and zeta potential measurements were conducted in 1 mM PBS pH 7.4 buffer using a Zetasizer Nano ZS (Malvern Panalytical, Malvern, Worcestershire, UK). The MTS assay absorbance values were evaluated at 490 nm *via* an Apollo 11LB913 microplate reader (Berthold, Bad Wildbad, Germany). The radioactivity of samples was measured using Wizard^®^ 2 automatic gamma counter (Perkin Elmer, Waltham, MA, USA) and an HPGe detector connected to a PC-based Multichannel Analyzer (MCA, Canberra).

Instrumental thin layer chromatography (iTLC) analyses were performed with the use of Storage Phosphor System Cyclone Plus (Perkin Elmer, Waltham, MA, USA), glass microfiber chromatography paper impregnated with silica gel (iTLC SG, Agilent Technologies, Santa Clara, CA, USA), and methyl alcohol (MeOH) as mobile phase. Analyses were performed in experiments of ^109^PdNPs dissolution in H_2_O_2_ as well as, during stability studies. For additional radiochemical yield evaluation, we centrifuged nanoparticles after each synthesis step and measured the activity of collected fractions.

### Radionuclides

^109^Pd was produced by thermal neutron (1–2 × 10^14^ n cm^− 2^ s^− 1^) irradiation of a natural palladium target (~ 3 mg, metal powder) or enriched ^108^Pd (> 99%) (~ 1 mg, metal powder) in the Maria nuclear reactor (Otwock-Świerk, Poland) for 7 h. Following an 8-hour cooling time, the radioactive palladium was dissolved in 200–400 µL of aqua regia (HNO_3_:HCl–1:3) and heated at 130 °C to get almost completely evaporated. The remaining nitrates were removed by dissolving the residues in 0.1 M HCl three times (100 µL) and heating at 130 °C until almost complete evaporation. Finally, the residual material was reconstituted in 1 mL of 6 M HCl resulting in the formation of H_2_PdCl_4_.

Since the neutron irradiation of the natural Pd target can result in the formation of ^111^Ag as an impurity in the reaction of ^110^Pd(n,γ)^111^Pd $\:\to\:$^111^Ag, it is essential to remove it from the solution before application (Das et al. 2012). The removal of ^111^Ag can be accomplished by precipitating it as AgCl using AgNO_3_ in the modified procedure reported by Das et al. (Das et al. 2012). Briefly, 100 µL of 0.1 M AgNO_3_ solution in 0.1 M HNO_3_ (20 mg/ mL) was added to 1 mL solution of PdCl_4_^2−^ in 6 M HCl. After 2 min, the AgCl precipitate was centrifuged (4600 rpm, 5 min), and the obtained supernatant was carefully separated from the precipitate AgCl through the pipette. followed by evaporation until complete drying. Subsequently, the procedure was repeated using deionized H_2_O. At last, the palladium was suspended in a 0.1 M HCl (100 µL). The activity and radionuclide purity of the obtained ^109^Pd were determined by gamma-ray spectrometry. The diluted solutions gained after radiochemical processing of the irradiated target were measured using an HPGe detector connected to a PC-based Multichannel Analyzer (MCA, Canberra). The 88 keV (3.67%) gamma peak emitted by ^109m^Ag was used for the estimation of the radioactivity of ^109^Pd. Nevertheless, due to the very low content of the ^111^Ag radionuclide, the silver removal procedure is unnecessary when using an isotopically enriched ^108^Pd target.

Iodine radionuclides, ^125^I (t_1/2_ = 59.5 d) and ^131^I (t_1/2_ = 8.01 d), which were utilized for comparative cytotoxicity studies, were obtained from the National Centre for Nuclear Research, POLATOM Radioisotope Centre (Świerk, Poland). The specific activity was > 600 GBq/mg and > 550 GBq/mg for ^125^I and ^131^I, respectively. Both radionuclides were supplied in an aqueous solution of sodium iodide (NaI) with a pH of around 10–12, adjusted using sodium hydroxide or sodium carbonate buffer.

### Synthesis of 5 nm palladium – PEG nanoparticles

Palladium nanoparticles (PdNPs) were synthesized according to the modified method described in the paper (Jung et al. 2017). Briefly, 5.3 mg of PdCl_2_ was dissolved in HCl (1.2 M, 500 µL) and stirred at room temperature. Following dissolution, the Pd solution was diluted by adding 53.5 mL of H_2_O. Subsequently, a freshly prepared NaBH_4_ solution (3.5 M, 5mL) was added to the mixture and stirred for 20 min. Finally, an aqueous solution of polyvinylpyrrolidone (PVP) (4.8 M, 21mL) was added, and stirring was continued for 1 h at room temperature. Considering the spherical shape, 5 nm diameter of the nanoparticles, and a Pd density of 12.02 g/cm^3^, the concentration of nanoparticles was calculated to be about 2.9 × 10^13^ PdNPs in 1 mL.

Radioactive nanoparticles were synthesized through the same procedure, using correspondingly smaller amounts of reagents. Both radioactive and non-radioactive PdNPs were stabilized by polyethylene glycol (PEG-COOH) chains using the following procedure: the synthesized PdNPs (5 nm) were combined with a 200 molar excess of HS–PEG–COOH (5 kDa) and stirred for 30 min. Afterwards, the nanoparticles were purified by centrifugation at 10,000 rpm for 10 min using Vivaspin 500 centrifugal filters with a 10,000 MWCO polyethersulfone (PES) membrane.

To conduct comparative experiments, the PEGylated Pd nanoparticles labelled with radionuclides ^125^I (Auger emitter) and ^131^I (β^−^ and γ emitter) were synthesized as well. To accomplish this, 10 mL of the previously obtained Pd-PEG NPs (5 nm) were redispersed in deionized water. Subsequently, either ^125^I or ^131^I was added (300MBq), and the reaction was kept at room temperature for 1 h with continuous stirring. At last, HS-PEG-COOH (MW = 5 kDa) was introduced to the solution of NPs with the desired excess, and the reaction was continued for the next 30 min.

### Stability studies of Pd-PEG nanoparticles

The colloidal stability of the PEGylated PdNPs dispersion in 10 mM PBS buffer was evaluated at 37 °C for 16 days. The aggregation tendency was examined by assessing hydrodynamic diameter and zeta potential variations using the Dynamic Light Scattering (DLS) technique. Due to technical limitations, the application of protein-containing solutions, such as serum, was not allowed.

### Studies on the recoil of ^109m^Ag from ^109^Pd nanoparticles and ^109^Pd-cyclam complex

To investigate the liberation of ^109m^Ag from ^109^PdNPs, they were incubated in both water and PBS buffer (1 mM). The initial radioactivity of the ^109^PdNPs solution was 4.8 × 10^4^ cpm. The nanoparticles were precipitated from the solution by adding 1 M NaCl and subsequent centrifugation (13 400 rpm, 40 s). To determine the released ^109m^Ag from NPs, the supernatant was immediately measured after separation using NaI (Tl) scintillation detectors at 15 s intervals. The measurement started 164 s after the precipitation of nanoparticles, which is comparable to four half-lives of ^109m^Ag.

For comparison, we examined a system in which ^109^Pd^2+^ was complexed with cyclam (1,4,8,11-tetraazacyclotetradecane; C_10_H_24_N_4_; 200.33 g/mol) to form a stable complex (log K_ML_=56.9 (Harrington et al. 2005)). In this case, the following original procedure for studies of liberation ^109m^Ag was applied: 10 µL of ^109^Pd-cyclam complex was added into a centrifuge tube containing 1 mL of NaCl solution, resulting in radioactivity of ^109^Pd-cyclam solution around 7.5 × 10^4^ cpm. Following that, 100 µL of 0.1 M AgNO_3_ solution in 0.1 M HNO_3_ (20 mg/ mL) was added, and the solution was promptly mixed using a vortex shaker. The formed AgCl precipitate was then centrifuged (13 400 rpm, 40 s). The obtained supernatant was carefully separated from the precipitate, and AgCl was dispersed in 1 mL H_2_O. The radioactivity measurement began 139 s after the precipitation of nanoparticles, i.e. after 3.5 half-lives of ^109m^Ag, and was measured successively at 15 s intervals.

### PdNPs dissolution in a highly oxidative environment

To verify the concept of the dissolution of PdNPs in elevated concentrations of H_2_O_2_, selected concentrations of hydrogen peroxide were prepared by diluting a stock solution (30%) with deionized water. Then, nanoparticles were dispersed directly in prepared H_2_O_2_ solutions and incubated for 24 h at 37^o^C. Afterwards, the radioactivity of ^109^PdNPs and the released free ^109^Pd^2+^ cations were measured using iTLC, as described above.

### Internalization studies

Internalization studies were conducted on HepG2 cells. Briefly, a total of 6 × 10^5^ cells/well were seeded into 6-well plates and incubated overnight at 37 °C and 5% CO_2_. Following this step, the cells were rinsed with PBS, and the main test compounds (1 mL) were added and incubated at 4 °C for 1 h to prevent any internalization. Subsequently, the medium was collected into the tubes as the unbound portion and replaced with 1 mL of fresh medium. The plates were then incubated (37 °C, 5% CO_2_) at different time points of 6, 18, and 24 h. To determine the membrane-bound fraction, cells were rinsed twice with glycine-HCl buffer (pH ~ 2.8; 0.05 M) for 5 min at 4 °C. Eventually, the internalized fraction was collected by lysing the cells with a solution of 1 M NaOH.

### Cytotoxicity studies

The MTS test was used to perform cytotoxicity assays on HepG2 cells (10^4^ cells per well in 96-well plates). The preparation procedure was analogous to that described for the internalization studies. Both non-radioactive (9–150 µg Pd/mL) and radioactive compounds (180 µg Pd/mL for 40 MBq/mL, 90 µg Pd/mL for 20 MBq/mL, 45 µg Pd/mL for 10 MBq/mL) were suspended in fully supplemented growing medium, and 100 µL per well was added for 24–72 h incubation. Afterwards, the medium was replaced with fresh medium (100 µL/well) prior to MTS reagent addition (20 µL/well). The percentage of metabolically active cells was assessed by the addition of CellTiter96^®^ Aqueous One Solution Reagent and the measurement of the absorbance at 490 nm.

### Double-strand breaks analysis

To assess the extent of DNA DSBs (double-strand breaks) induced by ^109^PdNPs treatment, staining of phosphorylated H2A histone family member X (γH2A.X) was conducted. HepG_2_ cells with a density of 2.5 × 10^5^ per well were seeded into six-well plates with five sterile glass coverslips per well (⌀ 12 mm, Thermo Fischer Scientific (Waltham, MA, USA) and then incubated overnight. After the removal of the medium, cells were treated with compounds at different concentrations (0–180 µg/mL, 0–20 MBq/mL, and 0-100 MBq/mL of Pd-PEG, ^109^Pd-PEG, and ^131^I-Pd-PEG NPs, respectively), and staurosporine (0.5 µM) as a positive control, followed by incubation for 4 and 24 h. The protocol used was similar to the one described in a previous report (Wawrowicz et al. 2023). For γH2A.X foci detection, the primary antiphospho-Histone H2A.X (Ser139) antibody, clone JBW301, was diluted to a ratio of 1:100 with blocking buffer (BB- 4% BSA in TBS) and 350 µL of that was added to each well and incubated overnight at 4 °C. The next day, the primary antibody was switched with an anti-mouse IgG secondary antibody conjugated with CFTM 633. The antibody was dissolved in a blocking buffer according to the manufacturer’s instructions. The cells were then incubated for two h at room temperature with mixing. Finally, cells were rinsed 3 times with water, followed by nuclei staining with Hoechst 33258. The imaging was performed using an FV-1000 confocal microscope (Olympus Corporation, Tokyo, Japan) with ex/mm maxima: 630/650 nm for CF633 and ex/em maxima: 352/454 nm for Hoechst 33258. The results were analysed using the Fiji 2.9.0 version.

### ^109^PdNPs effects on 3D tumor spheroid model

HepG2 cells (1 × 10^3^) were cultured on 96-well U-bottom ultra-low adherent plates (Corning^®^, Corning, NY, USA) with 200 µL of growing medium for seven days ahead of the experiment, as previously reported (Wawrowicz et al. 2023). During the incubation period, 100 µL of the medium was replaced with fresh medium every two days. After seven days, both radioconjugates and non-radioactive compounds were added into the growing medium (100 µL). All the images were taken and analyzed with a ZEISS AxioVert. A1 Microscope and ZEN 2.1 software (Zeiss, Jena, Germany).

### Statistical analysis

For statistical analysis, one-way ANOVA and t-Student tests were performed with GraphPad Prism v.8 Software (GraphPad Software, San Diego, CA, USA). The results are presented as mean ± SD. *p* values are presented as: (*) *p* ≤ 0.05, (**) *p* ≤ 0.01, (***) *p* ≤ 0.001, and (****) *p* ≤ 0.0001.

## Results and discussion

### Production of ^109^Pd

Due to a cross-section of 12.2 b (Boros and Packard 2019), large quantities of ^109^Pd can be produced in a nuclear reactor through the ^108^Pd(n,γ) reaction. Using natural palladium as a target material, after seven hours of irradiation at a flux of 1–2 × 10^14^ n cm^− 2^ s^− 1^, more than 500 MBq/mg of ^109^Pd was obtained. In the case of enriched 98% ^108^Pd target material, the obtained radioactivity exceeded 2 GBq/mg. In the gamma-ray spectrum of the irradiated natural target recorded before radiochemical processing, photo-peaks characteristic of ^109^Pd (22.1 and 24.8 keV) and ^109m^Ag (88.2 keV) can be seen. Additionally, small peaks from ^111^Ag (96, 245, and 342 keV) impurity formed in reaction ^110^Pd(n,γ)^111^Pd $\:\to\:$^111^Ag can be obse^r^ved. The impurity from ^103^Pd formed from the ^102^Pd(n,γ) reaction is negligible due to the low abundance of ^102^Pd (1%) and neutron low cross section. The separation of ^111^Ag by co-precipitation with AgCl was very effective, and the gamma-ray spectrum of the ^109^Pd solution after radiochemical processing did not show any gamma photo-peaks characteristic of ^111^Ag. This indicates that ^109^Pd was obtained with nearly 100% radionuclidic purity. The average radiochemical yield of ^109^Pd after the radiochemical separation of silver was found to be approximately 80%.

^109^Pd, which was obtained by neutron activation, was utilized in the synthesis of 5 nm radioactive PdNPs. It is estimated that the mass of one of the 5 nm nanoparticles is 7.9 × 10^− 18^ g, and it can be assumed that it contains 4.4 × 10^4^ Pd atoms, with 1–2 being radioactive. By using a high-flux reactor with a neutron flux of 10^15^ n/cm^2^/s and a longer irradiation time, the number of ^109^Pd atoms in the nanoparticle increases to 20–30.

### Synthesis and characterization of 5 nm Pd-PEG nanoparticles

Due to the strong affinity of thiol groups to palladium atoms, PVP molecules were completely displaced on the surface of nanoparticles. The synthesized PEGylated 5 nm (Pd-PEG NPs) are stable in solutions because the PEG molecules stopping layer on the PdNPs surface provides an electrostatic repulsion force resulting from the electric double layer.

The same procedure was followed to synthesize radioactive nanoparticles using ^109^PdCl_2_. As a result of the synthesis, ^109^PdNP-PEG–COOH nanoparticles with activity 10 MBq/mg (0.9 GBq/nmol NP) were obtained.

**Fig. 1:**
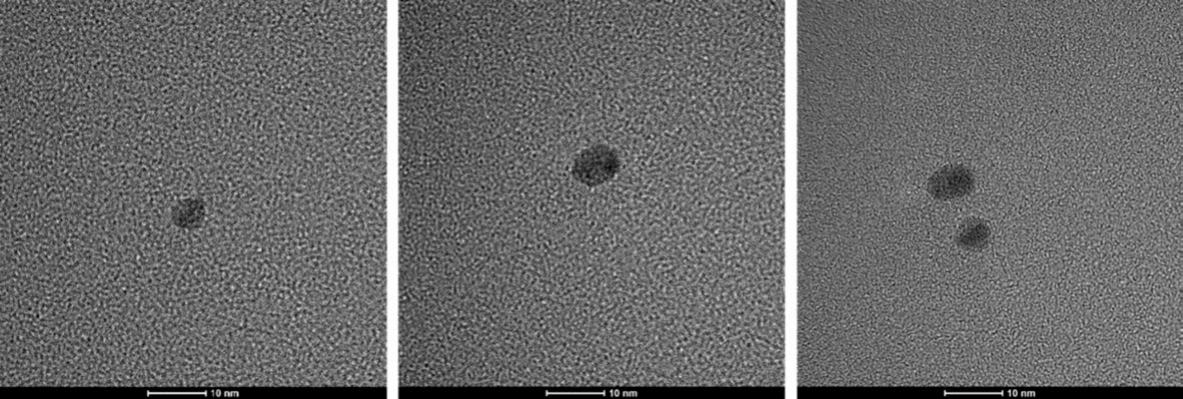
Transmission electron microscopy images of PdNP-PEG–COOH nanoparticle. Scale bars correspond to 10 nm

Synthesized PdNP-PEG–COOH nanoparticles were characterized using TEM and DLS methods. Figure 1 shows that the particles have a size of approximately 5 nm based on transmission electron microscopy. It was not possible to observe the “corona” around the Pd-NPs due to the poor interaction of the electron beam with the PEG molecules (low electron density), in contrast to the strong scattering of the electron beam when it interacted with metallic nanoparticles.

**Fig. 2:**
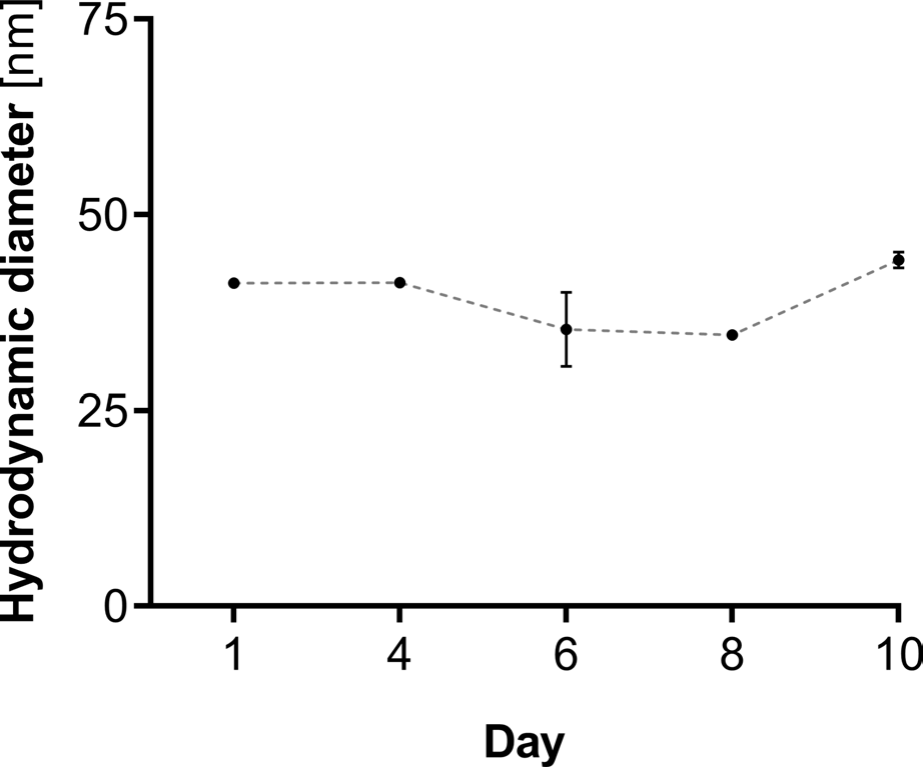
Changes in the hydrodynamic diameter of the Pd-PEG nanoparticles incubated in 10 mM PBS buffer

The hydrodynamic diameter of PEGylated PdNPs was around 40 nm, as determined by DLS in PBS solution, and was notably larger than the diameter measured by TEM. As both of the applied techniques are based on different approaches, within DLS it is possible to identify the polymer presence on the outer surface of nanoparticles, thus the diameter measured with this method directly reflects the PEG chains located on PdNPs surface, leading to increased size. The zeta potential of PdNP-PEG-COOH was − 19.9 ± 1.57 mV and the negative value of this potential indicates that the particles repel each other, contributing to the considerable stability of the colloid. Moreover, negative surface charge makes them suitable candidates for liver treatment by preventing kidney accumulation. As shown in Fig. 2, the particles do not tend to aggregate until 10 days, which was confirmed by the lack of changes in hydrodynamic diameter during a whole experimental assessment.

### Liberation of ^109m^Ag from ^109^Pd nanoparticles

When using in-vivo generators in nuclear medicine, it is important to consider the behavior of the daughter radionuclide after radioactive decay. In our previously published paper, we studied liberation of ^109m^Ag from core-shell Au@^109^Pd nanoparticles (Gharibkandi et al. 2023). Current research aimed for deeper investigation of this topic and comparison the behavior of the ^109m^Ag daughter radionuclide after the radioactive decay of ^109^Pd on ^109^PdNPs (5 nm) and in the ^109^Pd-cyclam complex.

**Fig. 3:**
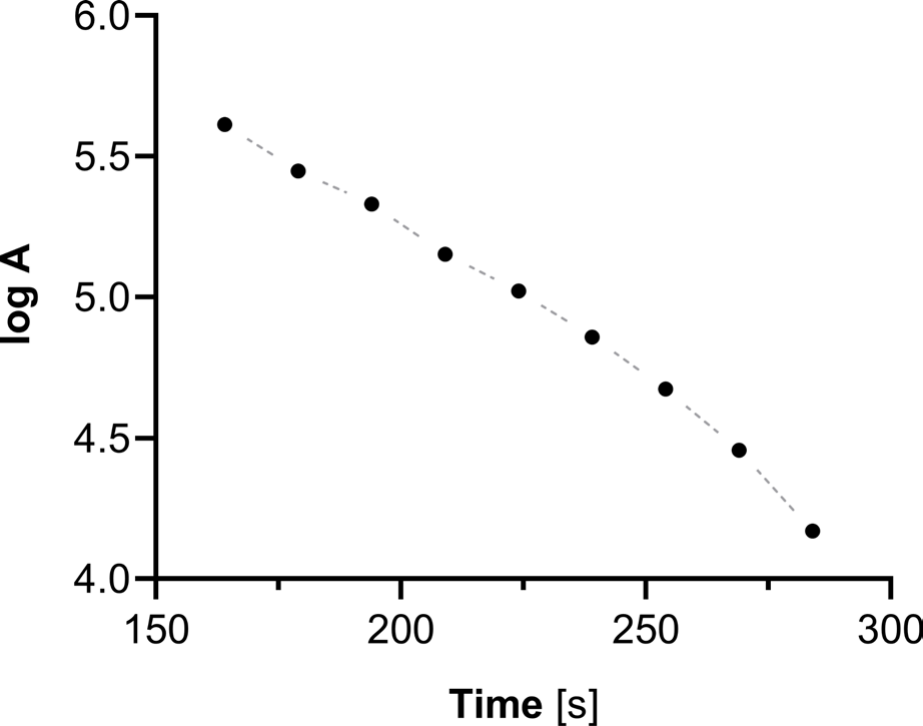
Decay curve of AgCl precipitated from the ^109^Pd-cyclam complex solution. The measurement started 2 min and 44 s after the addition of AgNO_3_, which corresponds to four half-lives of ^109m^Ag. The radioactivity of the AgCl was measured successively at 15-second intervals. Activity in cpm

Our initial studies, which involved binding of ^109^Pd to a biomolecule using the macrocyclic cyclam, demonstrated the complete release of ^109m^Ag from the complex. Figure 3 illustrates the decay curve of ^109m^AgCl precipitated from the solution of the ^109^Pd-cyclam complex. By extrapolating the activity to time zero, we observed that the estimated activity of ^109m^Ag is equivalent to the activity of ^109^Pd in the complex. Therefore, despite its short half-life of 39 s, ^109m^Ag can diffuse away from the target site and affect healthy cells.

**Fig. 4:**
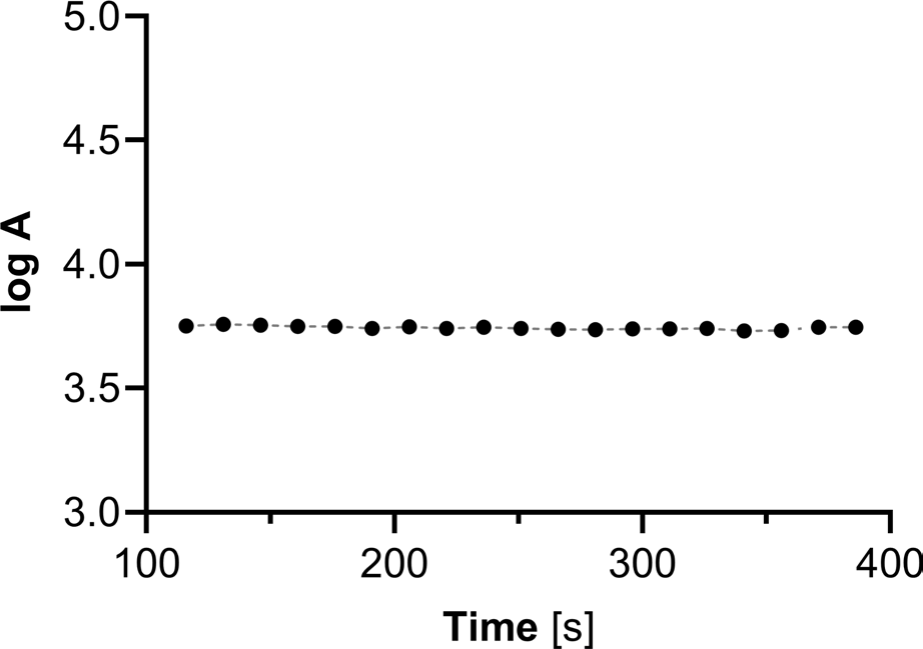
Activity of the supernatant after precipitation of the nanoparticles by 1 M NaCl solution. The measurement started 116 s after the addition of NaCl, which corresponds to three half-lives of ^109m^Ag. The radioactivity of the supernatant was measured successively at 15-second intervals. Activity in cpm

To prevent the diffusion of ^109m^Ag from the target site, we propose a solution involving the use of a ^109^Pd/^109m^Ag in-vivo generator in the form of 5 nm ^109^Pd nanoparticles. In contrast to chelator-based in-vivo generators, we found complete retention of ^109m^Ag on Pd nanoparticles. While the parent radionuclide is incorporated into a metallic nanoparticle instead of a chelate complex, we do not observe the liberation of ^109m^Ag from the nanoparticles, as shown in Fig. 4. The constant level of radioactivity is attributed to the presence of nanoparticle residues after a short centrifugation time (13400 rpm, 40 s.).

### ^109^PdNPs dissolution in a highly oxidative environment

**Fig. 5:**
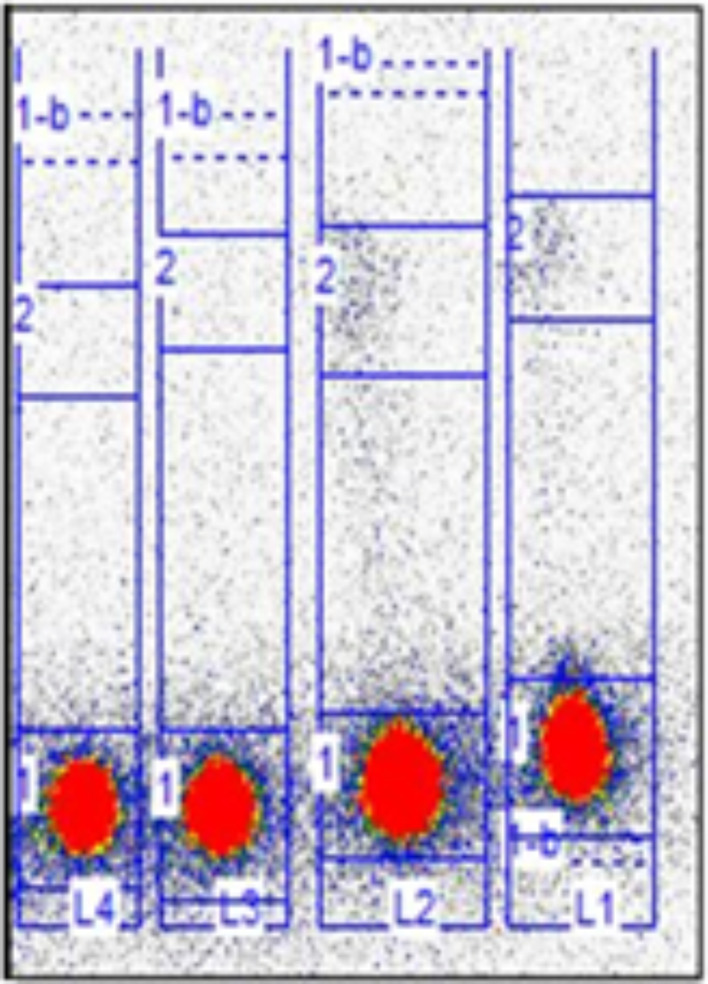
The iTLC strips illustrate the lack of dissolution of 109PdNPs in H2O2 solutions. L4 strip is for concentration H_2_O_2_ 10 mM, L3 for 10µM, L2 for 10 nM and L1 for human serum. Incubation time 24 h at 37^o^C

To verify the concept of PdNPs dissolution in HepG2 cells with elevated H_2_O_2_ concentrations, as described in the introduction, we conducted studies of the behavior of PdNPs in a wide range of H_2_O_2_ concentrations. The concentration of H_2_O_2_ in normal cells is generally in the nanomolar range (Sies 2017). Therefore, we launched our investigations at a concentration of 10 nM and implemented also higher concentrations of up to 10 mM. Figure 5 shows iTLC strips for different H_2_O_2_ concentrations. If the ^109^PdNPs nanoparticles were dissolved, we would observe migrating ^109^Pd^2+^ cations with the solvent front. However, for all of the tested H_2_O_2_ concentrations, radioactivity remained at the site of application. This indicates the lack of dissolution of ^109^PdNPs, even in solutions with high H_2_O_2_ levels.

### Internalization of ^109^Pd-PEG nanoparticles

**Fig. 6:**
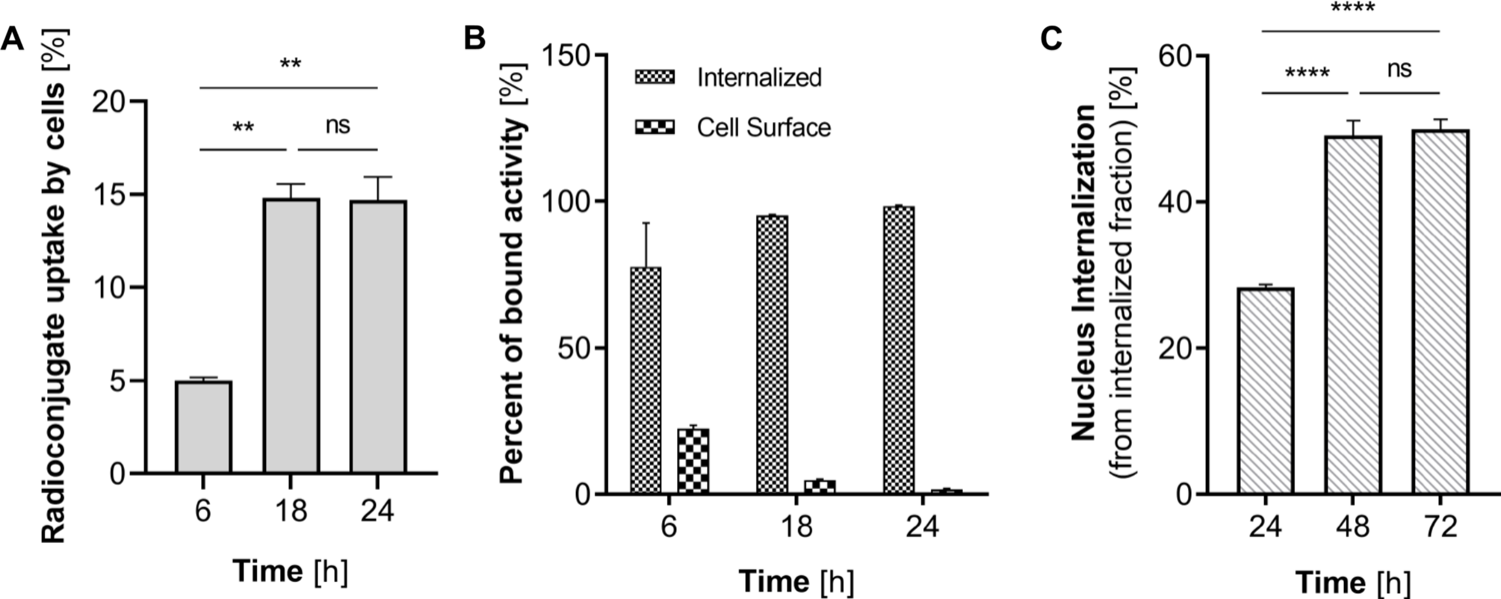
^109^PdNPs bound to HepG2 cells (**A**); percent of bound activity that was later internalized to the cells (**B**); intranuclear uptake of synthesized radioconjugates (**C**)

Internalization to the cell nucleolus or localization on the cell membrane is crucial for effective Auger electron radionuclide therapy (Ku et al. 2019b). As investigated NPs do not undergo even partial dissolution in highly oxidative environment, we aimed to verify whether, due to their small diameter, they are able to passively penetrate the nuclear membrane. The performed studies using radioactive ^109^Pd nanoparticles revealed that the nanoparticles accumulated inside the cells in a time-dependent manner. Radioconjugate uptake, reaching approximately 5% after six h, was significantly (*p*$\:\le\:$ 0.01) enhanced after 18 h (~ 15%) and maintained almost unchanged (*p* = 0.9379) after 24 h (Fig. 6A). As shown in Fig. 6B, over 75% of the total bound fraction was subsequently internalized starting from first investigated time point. This process progressed over time, leading to over 95% and 98% internalization after 18 and 24 h, respectively. Therefore, our studies showed that PEGylated nanoparticles can successfully penetrate the HCC cell membrane without the need for internalizing vectors.

In our subsequent studies, we aimed to determine the proportion of Pd internalized into the nucleus from the cell cytosol. To assess the efficiency of internalization, we extracted cell nuclei fractions from HepG2 cells that were treated with ^109^Pd-PEG NPs and measured the intracellular ^109^Pd radioactivity after 24, 48, and 72 h of incubation (Fig. 6C). Approximately 28% of the internalized ^109^Pd in the cytosol was efficiently transferred to the nucleus after 24 h. This process significantly increased to almost 50% after 48 h (*p*$\:\le\:$ 0.0001).

As it is well-known, the nucleus is separated from the cytosol by the double nuclear membrane, which contains 2000 to 5000 specialized channels called nuclear pore complexes through which transport to and from nucleus occurs. The 5 nm Pd-PEG nanoparticles are hydrophilic and thus able to passively diffuse into the cell nucleus, as confirmed by Fig. 6C and previously discussed research. Due to the presence of radioactive ^109^Pd-PEG nanoparticles in the nucleus, it was expected to effectively increase its cytotoxicity through the interaction of Auger electrons or formed oxygen radicals with DNA.

### In vitro toxicity

**Fig. 7:**
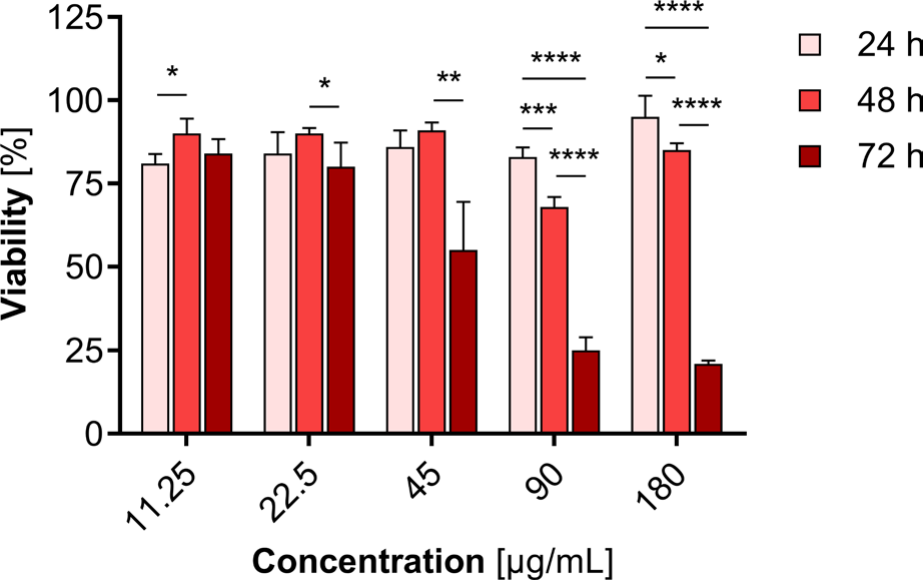
Metabolic activity of HepG2 cells after treatment with different concentrations of nonradioactive Pd-PEG after 24 h 48 h and 72 h

The viability of HepG2 cells incubated with non-radioactive Pd-PEG-NPs and ^109^Pd-PEG-NPs was evaluated using the MTS assay. Nonradioactive Pd–PEG bioconjugates were used with various concentrations 11.25 µg/mL (3 × 10^− 3^ nmol/mL NP) to 180 µg/mL (0.05 nmol/mL NP). These studies aimed to investigate whether non-radioactive Pd-PEG can lead to mitochondrial dysfunction and cell death. As shown in Fig. 7, almost no significant reduction in mitochondrial activity was found with doses up to 45 µg/mL of Pd-PEG NPs. In higher concentrations, the viability of cells gradually decreased with the increase of Pd concentration, reaching below 25% viability at the remarkably high concentration of 180 µg/mL. It is important to note that the most meaningful changes were identified after 72 h. Similar results were obtained by Rajakumar et al.(Rajakumar et al. 2015) studying 60 nm PdNPs on HepG2 cells. Interestingly, the toxicity levels were found to be similar to that of cationic Pd^2+^. The comparison between the cytotoxicity of PdNPs and 2 nm platinum nanoparticles and 30 nm core shell Au@PtNPs on HepG2 cells indicated slightly higher cytotoxicity induced by PdNPs compared to both 2 nm PtNPs and 30 nm Au@PtNPs (Wawrowicz et al. 2022).

In the cytotoxicity studies of radioactive ^109^Pd-PEG, we used nanoparticles with radioactivities ranging from 11.25 MBq/mL to 100 MBq/mL. In these experiments, the concentration of PdNPs varied from 22.5 µg/mL to 180 µg/mL. Figure 7 shows that if the mass concentration exceeds 22.5 µg/mL, PdNPs may exhibit chemical toxicity in addition to radiotoxicity, especially after 72 h of incubation.

**Fig. 8:**
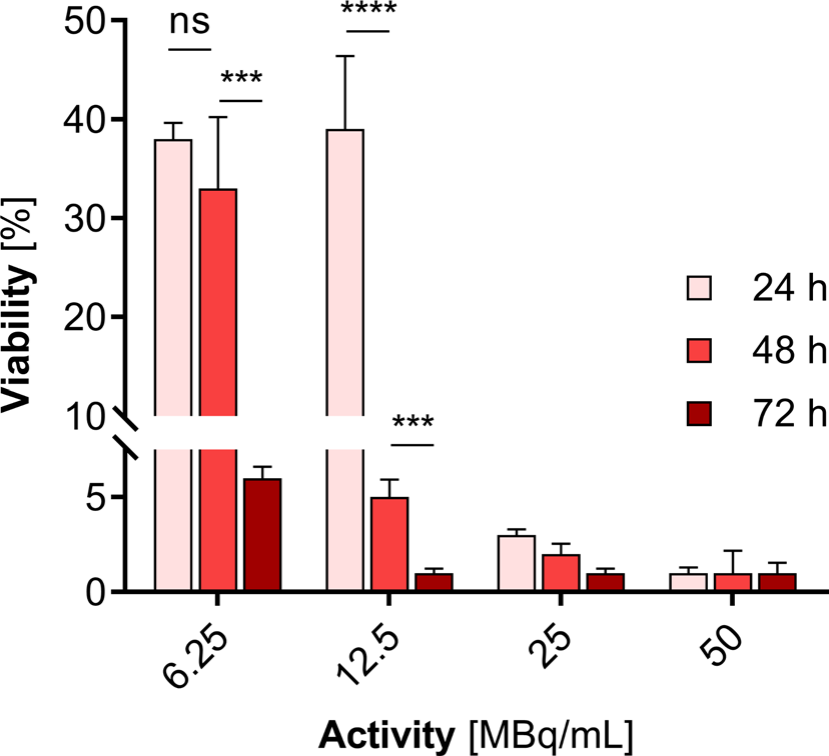
Metabolic viability of HepG2 cells after treatment with different radioactive doses of ^109^Pd-PEG after 24 h, 48 h, and 72 h treatment

We observed a significantly stronger cytotoxic effect when the radioactive ^109^Pd-PEG interacted with HepG2 cells than nonradioactive compounds. Figure 8 illustrates the dependence of cell viability on the radioactivity of ^109^Pd-PEG. As demonstrated, toxicity in a dose-dependent manner, progressing over time was observed. Furthermore, even at the lowest radioactivity level of 6.25 MBq/mL ^109^PdNP-PEG, a significant inhibition of cell metabolic activity was observed (6% after 72 h, *p*$\:\le\:$ 0.001). Extended incubation resulted in a constant reduction in mitochondrial activity, leading to almost complete cell death with < 5% of unaffected mitochondrial function at 25 MBq/mL. By comparing the data presented in Figs. 7 and 8, it is obvious that the cytotoxic effect caused by the chemical generation of reactive oxygen species (ROS) is negligible compared to the radiotoxic effect presented in Fig. 8.

As we discussed earlier, the ^109^Pd/^109m^Ag in-vivo generator, similarly to ^161^Tb, emits together β^−^ particles and Auger electrons. To verify the superior therapeutic efficacy with simultaneous emission of β^−^ particles, conversion, and Auger electrons in comparison with using β^−^ or Auger electron emitters with the same activity alone, we performed cytotoxicity experiments using Pd nanoparticles labeled with ^125^I (Auger electrons, γ emitters) and ^131^I (β^−^, γ emitters) radionuclides.

**Fig. 9:**
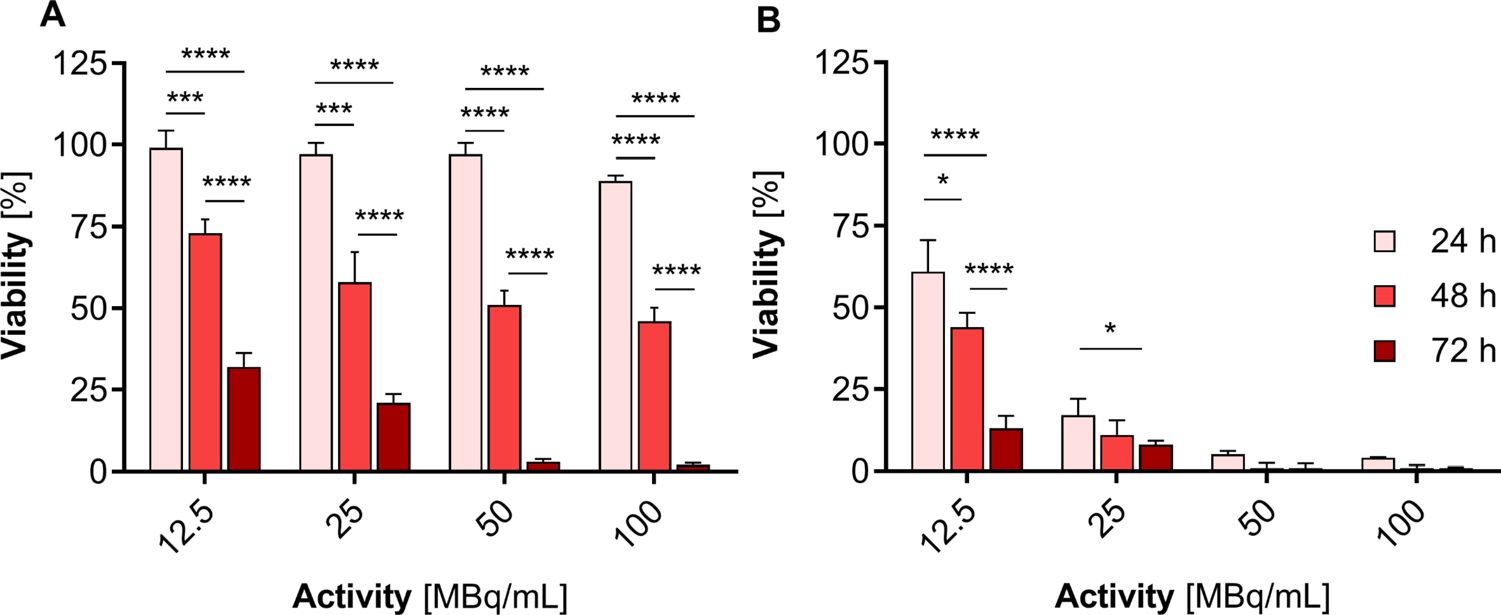
Metabolic viability of HepG2 cells after treatment with different radioactive doses of ^125^I-Pd-PEG (**A**) and ^131^I-Pd-PEG (**B**) radio conjugates

It was possible to attach ^125^I and ^131^I to the surface of Pd NPs by exploiting the strong affinity between noble metals and iodine atoms. Figure 9 presents the results of MTS tests conducted on HepG2 cells using both ^131^I-Pd-PEG and ^125^I-Pd-PEG radioactive NPs. Our findings indicate that PdNPs labeled with ^109^Pd, despite its shorter half-life and the smallest number of radioactive decays during cytotoxicity examination, were significantly more cytotoxic than those labeled with either ^131^I or ^125^I. We observed a similar effect in cytotoxicity studies of the Au@^109^Pd core–shell nanoparticles conjugated to trastuzumab, which showed greater efficacy compared to ^198^AuNPs-trastuzumab (emitting β^−^ particles) (Gharibkandi et al. 2023). These findings prove that the therapeutic efficacy of medium-energy β electrons can be significantly improved by adding short-range Auger electrons, as is in the case with ^161^Tb-based radiopharmaceuticals.

It should be noted that the comparison of cytotoxicity between the data presented in Figs. 8 and 9 is only an approximation because the half-life of the radionuclides and the energy have not been considered. Furthermore, the emitted γ radiation was neglected due to its insignificance in comparison to corpuscular radiation. However, the obtained results clearly demonstrate that the cytotoxicity of ^109^Pd - a mixed radiation emitter (β^-^, Auger) is much greater than that of Auger electron and β^-^ radiation emitters, assuming the same activities.

### Radiotoxicity studies on 3D tumor spheroid model

**Fig. 10:**
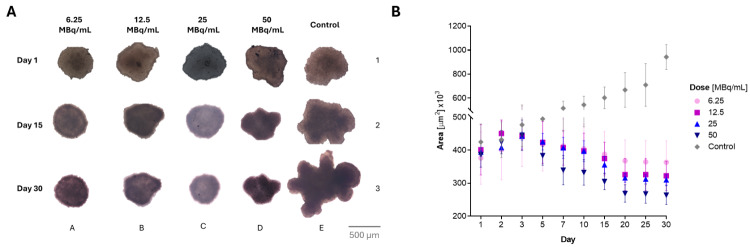
Effects of ^109^Pd-PEG radioconjugates against HepG2 tumor spheroid model. (**A**). Microscope images of representative spheroids - structures extracted from the background due to differences in contrast. All photos were taken using a 10X lens. However, the last photo (panel 3D) was taken using a 4X objective due to the high increase in the spheroid size of the control group and subsequently reconstructed; (**B**) Time dependence spheroids growth

In contrast to monolayer cultures, three-dimensional (3D) cell cultures better mimic tissue physiology and exhibit the characteristics of poorly perfused tumors. These models are suitable for evaluating the effectiveness of anticancer drugs (Białkowska et al. 2020). Hence, we also conducted cytotoxicity studies on spheroids formed from HepG2 cells incubated with ^109m^Pd-PEG nanoparticles. In Fig. 10 we present microscopic images of the spheroids treated with ^109^Pd-PEG NPs and changes in tumor areas continuously measured for 30 days. Remarkable surface changes were observed in the spheroids of both treated and control samples. The area of the control samples increased ~ 2.5 times during the 30 days of observation. In the treated samples, the initial signs of tumor growth inhibition were observed 72 h after NPs injection. Despite the relatively short half-life of ^109^Pd (13.7 h), the growth of spheroids was inhibited until day 30 of the study. For all tested concentrations, inhibition of tumor growth was found. However, we also observed the shrinkage of spheroids for samples treated with higher activities of 25 MBq/mL and 50 MBq/mL.

### DNA double-strand breaks (DSBs)

Damage to the genetic materials is considered as one of the most important effects in radionuclide therapy. Single-strand breaks (SSBs) and especially double-strand breaks (DSBs) are the two primary and most desired damages that can occur in the DNA molecule. These breaks can occur through direct ionization of the DNA caused by ionizing radiation (direct effects) or by the interaction of reactive oxygen radicals generated from water with the DNA strand (indirect effects). In our study, we compared the induction of DSBs following exposure to β^−^ radiation emitted from ^131^I-PdNPs and β^−^ and Auger electrons emitted by ^109^Pd. The phosphorylation of the H2A.X occurs at one of the initial stages in the DSB repair pathway. As a result, the scoring of phosphorylated histone (γH2A.X) foci is widely utilized to quantify DSBs. H2A.X phosphorylation happens at the site of DSB immediately after its formation and can be seen under a microscope as distinct foci after antibody labeling as described before (Hernández et al. 2013).

**Fig. 11:**
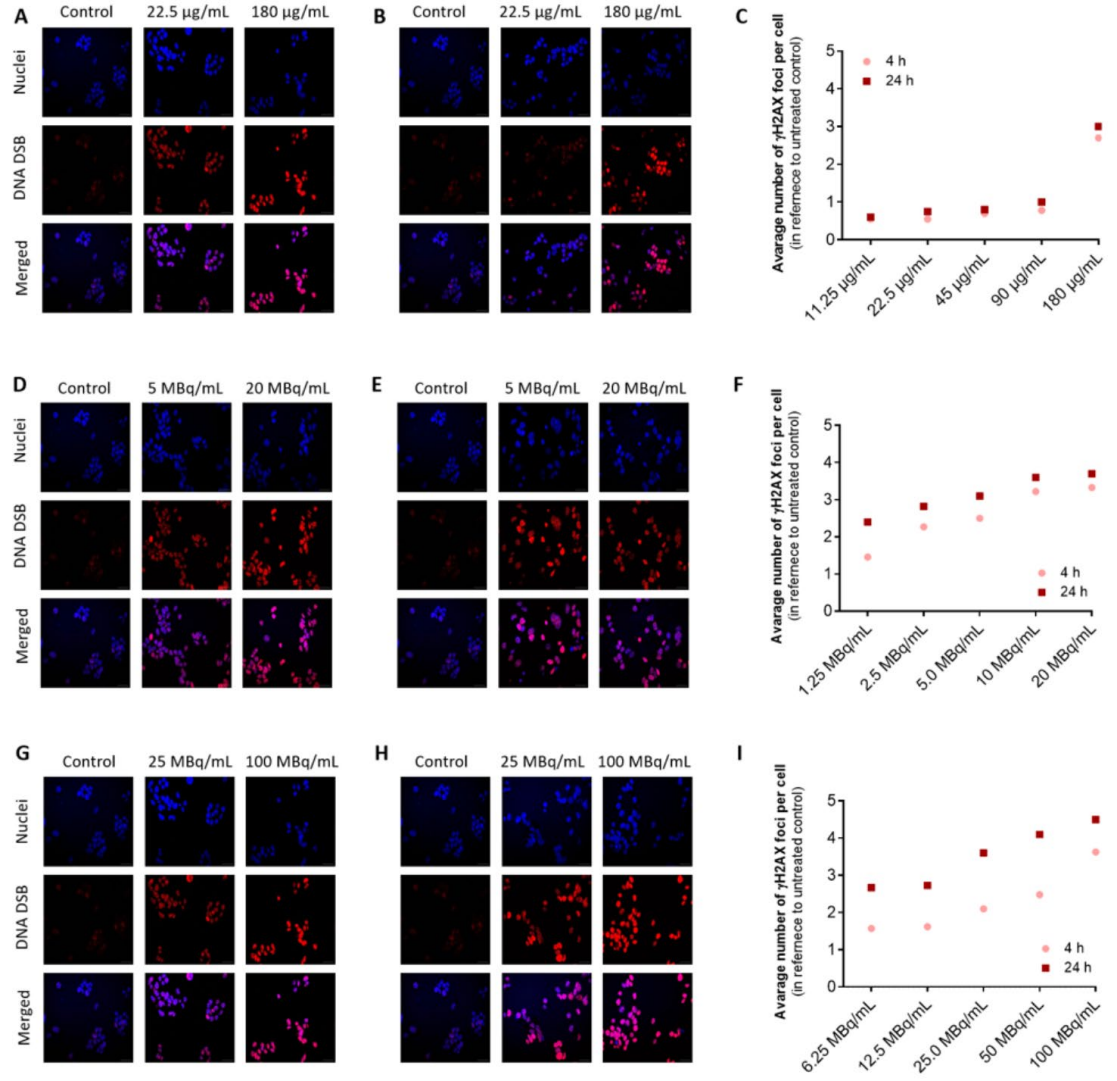
γH2A.X foci formation in HepG2 cells after treatment with Pd-NPs (**A**-**C**), ^109^Pd-NPs (**D**-**F**), and ^131^I-Pd-NPs (**G**-**I**). Panels A, D, and G – imaging 4 h post-treatment; panels B, E, and H – imaging 24 h post-treatment. Images only of selected compounds doses are shown

DNA DSBs found in HepG2 cells visualized with γH2A.X foci after treatment with non-radioactive Pd-PEG NPs (A-C), radioactive ^109^Pd-PEG (D-F), and ^131^I-Pd-PEG NPs (G-I) are presented in Fig. 11.

A growing number of DSBs cells was found after treatment of HepG2 cells with ^109^Pd-PEG NPs starting from radioactivity 1.25 MBq/mL. In the case of ^131^I-Pd-PEG nanoparticles, DSBs formation is observed starting from a concentration of 6.25 MBq/mL, with a much smaller DSBs number occurring. Considering the redox activity of PdNPs, we could also expect an increased DSB ratio in cells after treatment with the nonradioactive compound. It is widely reported that ROS may induce severe DNA damage, also including DNA DSBs. However, as can be seen in Fig. 11, in the concentration range up to 90 µg/mL, the number of DSBs remains at the level of the control sample. Only at a concentration of 180 ug/mL we identified a significant number of γH2A.X foci both after 4 h and 24 h.

## Discussion

When using in-vivo generators it is crucial to assess whether after radioactive decay, the daughter radionuclide remains in the bioconjugate or diffuses out of initial structure. There are two opportunities where the daughter can detach from the parent radionuclide: either due to elemental differences between them or as a result of the physical and chemical disturbances caused by the nuclear decay process. In many cases, the chemical change of atomic number (as a result of nuclear decay) is sufficient to induce a difference between parent and daughter chemistry. This occurs for example in the decay of ^131^I to noble gas ^131^Xe, when Xe atoms escape from ^131^I labeled molecules. However, even when the parent and decay product have nearly identical chemical behavior, as with transitions between two lanthanides, there is still a possibility for a chemical change due to the atomic effects of nuclear decay (Edem et al. 2016). Generally, if the nuclear transition recoil energy of the daughter radionuclide exceeds the binding energy in the complex, breaking of the bonds and the daughter radionuclide escape from the original structure can be expected. Alpha transition is one of examples where the recoil energy significantly exceeds the energy of chemical bonds, and the complete release of the daughter radionuclides from the molecules is observed. However, for other decay types, this process is more complex. In a series of articles Szücs, Zeevaart et al. described chemical consequences that may occur in the case of beta, electron capture, and internal transition decay (van Rooyen et al. 2008; Zeevaart et al. 2012b; Zeevaart et al. 2012a). In most β^−^ decays, where beta and neutrino particles are emitted, the recoil energy imparted to the daughter does not exceed a few eV and is not sufficient to displace the daughter from strong multidentate chelates. For example, in the case of the ^90^Sr/^90^Y complexed pair with DOTA, it was shown that only 1% of all β^−^ decays led to the release of the ^90^Y daughter (Zeevaart et al. 2012b).

The situation changes significantly when β^−^ decay is accompanied by the emission of Auger electrons, as seen in the ^166^Dy/^166^Ho in-vivo generator. It has been found that following the β^−^ decay of ^166^Dy, an excited state of ^166^Ho* is formed. The de-excitation of ^166^Ho* occurs *via* internal conversion instead of γ emission and energy is transferred to the electrons of the inner shell, resulting in the creation of electron vacancies. Electrons from the outer shells are organized to fill the vacancies, emitting an Auger electron cascade through the created ^166^Ho atom. As a result, the de-excited daughter radionuclides become highly charged which leads to electron uptake from the surrounding chelator donor atoms. Moreover, due to the electron transfer to highly charged atoms, donor atoms of chelators acquire a positive charge. The metal-ligand bonds are then broken as a result of the repulsive force between the positively charged atoms, and the daughter ^166^Ho is released as free cations (Wang et al. 2022).

A similar situation occurs in the case of the proposed in our work ^109^Pd/^109m^Ag in-vivo generator, which combines β^−^ emission from the parent ^109^Pd radionuclide with a high emission of Auger electrons from the daughter ^109m^Ag. In the case of the ^109^Pd/^109m^Ag macrocyclic complexes, an additional factor contributing to the release of the daughter radionuclide is the high chemical difference between Pd^2+^ and Ag^+^ cations. Furthermore, tetraaza macrocyclic ligands exhibit very weak binding of Ag^+^ ions - K_ML_ of Pd^2+^cyclam complex is 56.9 (Harrington et al. 2005), whereas for a very similar Ag^+^ cylcen it is only 6.6 (Rajakumar et al. 2015) .

Interestingly, in the case of ^109^Pd-PEG nanoparticles used in our studies, the release of ^109m^Ag is not observed. As it is well recognized that the metallic phase contains several delocalized electrons, after the nuclear decay of ^109^Pd, the highly positively charged daughter ^109m^Ag radionuclide extracts the delocalized electrons from the Pd nanoparticles. As a result, the positive charge is rapidly transferred to the entire nanoparticle, causing only a negligible change in the whole nanoparticle charge. Consequently, the release of ^109m^Ag from the nanoparticles is not achievable. This same effect was also observed by Wang et al. (Wang et al. 2022) in their studies of a ^166^Dy/^166^Ho in-vivo generator with radionuclides deposited on the AuNPs surface. This phenomenon is, of course, beneficial in terms of maximizing therapeutic effectiveness. As ^109m^Ag remains within the structure of the NPs, there is negligible risk of ^109m^Ag unspecific localization in different non-target tissues after treatment. Hence, it significantly reduces the risk of post-treatment side effects.

In order to achieve optimal treatment results, it is crucial to deliver Auger-electron-emitting radionuclides to the cell nucleus, preferably close to the DNA. As previously mentioned, this can be achieved by dissolving the small platinum NPs in the cytoplasm of HepG2 cells, which is commonly known to be a cancer cell line with an increased redox potential in the cytosol (Shoshan et al. 2019; Szatrowski and Nathan 1991). In our work, we intended to investigate whether the described effect of dissolving platinum nanoparticles also applies to PdNPs. If such a phenomenon occurred, easy transport of ^109^Pd ions from the cytoplasm to the cell nucleus and incorporation into DNA would be possible, favouring DNA damage and therapeutic response. However, as shown in Fig. 5, Pd-PEG NPs did not dissolve, even when exposed to high concentrations of hydrogen peroxide. Despite this, both non-radioactive and ^109^Pd-PEG nanoparticles exhibited high toxicity levels during our studies. This relates to the significant internalization of Pd-PEG NPs into the cytoplasm, followed by their ~ 50% cell nucleus uptake through the nuclear pore complex. Nuclear pore complexes allow the passive diffusion of ions, small molecules and nanoparticles through aqueous channels with a diameter of ∼9 nm (Panté and Kann 2002). The main condition for transportation is that the substances being transported must be hydrophilic (Ma et al. 2012). Therefore, the observed toxicity of non-radioactive Pd-PEG NPs on HepG2 cells could be associated with the catalytic decomposition of H_2_O_2_ on the surface of PdNPs, resulting in the formation of reactive hydroxyl radicals or singlet oxygen molecules. Of course, for ^109^Pd-PEG NPs, the radiotoxic effect of β^−^ particles and Auger electrons emitted in the cell nucleus predominates.

In our studies, we have demonstrated that Pd-PEG NPs labelled with ^109^Pd have a significantly higher cytotoxic effect on HepG2 cells compared to labelled with ^131^I (beta emitter) and ^125^I (Auger electron emitter). This effect has been also observed repeatedly when comparing bioconjugates labelled with ^161^Tb and ^177^Lu. As explained, the higher efficacy of ^161^Tb compared to ^177^Lu and other β^−^ radioisotopes like ^47^Sc and ^67^Cu is mainly due to the larger amount of Auger and low-energy conversion electrons, whose doses are deposited over relatively short distances (Champion et al. 2016). The authors of the study demonstrated that in a 100-µm metastasis, CE and Auger electrons were responsible for 71% of the radiation dose deposited by ^161^Tb. This is in contrast to almost pure β^−^ emitters, where more than 99% of the absorbed energy is due to β^−^ particles for all spheres. For tumors with a 5 mm diameter, the absorbed dose was similar across all three radionuclides. As shown in Figs. 8 and 9 we observed a similar situation when we compared the cytotoxicity of ^109^Pd-PEG with that of ^131^I-labeled Pd-PEG NPs. Our experiments conducted to determine cytotoxicity involved examining layers of cell colonies (1–2 layers, 10–20 μm) and spheroids with a diameter of ~ 1 mm. In the case of a cell layer, we found that metabolic activity is significantly reduced to 25% for 12.5 MBq/mL. However, for 1 mm spheroids that limit metastases, the effect is not as significant and is limited to inhibiting spheroid growth or slightly decreasing their surface area. The toxicity of ^109^Pd-PEG NPs for spheroids is comparable to that of ^198^Au nanoparticles x017B;elechowska-Matysiak et al. 2023) which emit β^−^ radiation with an energy of β_max_ = 961 keV, a similar energy to that emitted by ^109^Pd. However, unlike ^109^Pd, ^198^Au does not emit Auger electrons. Thus, the advantages of ^109^Pd-PEG become visible in the case of small cancer metastases. However, the β radiation component allows for the parallel destruction of larger cancer lesions Therefore, ^109^Pd-PEG nanoparticles, similar to ^161^Tb radioconjugates, should be intended for applications in metastatic cancers.

Cytotoxicity results of ^109^Pd-PEG and ^131^I-Pd-PEG NPs directly correlate well with the occurrence of DSBs in the HepG2 cells visualized by γH2A.X foci. The high LET Auger and conversion electrons emitted by the ^109^Pd/^109m^Ag in-vivo generator are responsible for the high number of DSBs formation. The number of DSBs is significantly reduced following β^-^ particle irradiation, attributable to the extended interaction range and lower LET of β^-^ particles (Faraggi et al. 1994). The significance of DSBs in radionuclide therapy was demonstrated by Tounekti et al. (Tounekti et al. 2001) through their studies on Chinese hamster fibroblasts. They found that cell death may occur in the form of apoptosis if there are more than 150 000 SSBs and only 500 DSBs. However, if the number of SSBs and DSBs is less than 150 000 and 500, respectively, cell death does not occur; instead, a reparative process emerges. This information is essential for understanding the effects of radiation on cells during radionuclide therapy, which aims to selectively target and destroy cancer cells while minimizing damage to healthy tissues.

## Conclusions

Nanostructures have been proposed as a novel approach for the treatment of HCC that is more effective than traditional methods such as sorafenib, transarterial chemotherapy, and radioembolization. The application of nanostructures can reduce therapeutic toxicity and facilitates more precise targeting of the affected area. In this paper, we discuss the use of ^109^Pd-PEG nanoparticles as an in-vivo ^109^Pd/^109m^Ag generator. The studies revealed that these nanoparticles were significantly more effective in-vitro than Pd-PEG NPs labeled with either ^125^I (Auger emitter) or ^131^I (β^-^ emitter). This is due to the unique potential presented by the ^109^Pd/^109m^Ag in-vivo generator, which emits both β^-^ and conversion/Auger electrons.

As presented in the introduction, we expected the dissolution of PdNPs in H_2_O_2_ solution, the concentration of which is elevated in HepG2 cells. This would allow to Auger electrons emitted from ^109^Pd^2+^ to interact directly with the DNA strand. However, studies carried out in a wide range of H_2_O_2_ solutions have shown that this process does not occur. On the other hand, the approximately 50% accumulation of 5 nm ^109^Pd-PEG nanoparticles in the cell nucleus caused a significant cytotoxic effect. The nucleus localization of high-activity Auger emitters can lead to DNA strand damage through direct interaction with conversion electrons, higher energy Auger electrons, and ROS radicals generated within the nucleus. The interaction of β^-^ particles emitted by ^109^PdNPs localized in the nucleus with DNA is limited due to their low LET value. However, β^-^ particles can interact with DNA in neighboring cells, expanding the range of cytotoxic effects. Therefore, radiobioconjugates labeled with the ^109^Pd/^109m^Ag radionuclide generator, like ^161^Tb, might show the advantages of both β^-^ emitters (a few millimeters range of radiation, crossfire effect) and Auger electrons (large LET, double-stranded DNA breaks).

The proposed solution also provides another advantage: the accumulation of inorganic nanoparticles in the liver when introduced in-vivo. Typically, this is a major drawback that prevents the therapeutic use of nanoparticles in medicine. However, in the case of HCC tumors, it might be advantageous because it will enable the accumulation of ^109^Pd-PEG NPs in the targeted organ, where due to the leaky vasculature of HCC tumors (EPR effect), we can expect the accumulation in the cancer cells. In our future studies, we plan to incorporate targeting vectors such as glycyrrhetinic acid, transferrin, folate, or P-glycoprotein 1 (CD44) ligands to specifically target HepG2 cells while avoiding normal liver cells.

In recent years, there have been numerous studies on glypican-3 (GPC3), a highly expressed cell surface antigen found in about 75% of HCC cases. It was also found GC33 (codrituzumab), a humanized monoclonal antibody, has been shown to be specific for GPC3 antigen (Haruyama and Kataoka 2016). Additionally, preclinical studies conducted in the last five years have demonstrated that the α and β^-^emitters labeled GC33 antibody effectively and selectively reduced liver tumor volume in a mouse model (Bell et al. 2021). Consequently, in our future studies with ^109^Pd, we plan to incorporate GC33 as a targeting vector to specifically target HepG2 cells while avoiding normal liver cells.

## Data Availability

Data is available upon reasonable request to the corresponding author.
